# Prediction method for the tension force of support ropes in flexible rockfall barriers based on full-scale experiments and numerical analysis

**DOI:** 10.1038/s41598-024-60508-6

**Published:** 2024-04-30

**Authors:** Xin Qi, Lei Zhao, Qing-Cheng Meng

**Affiliations:** 1 School of Civil Engineering, Southwest Jiaotong University, Chengdu, China; 2 School of Civil Engineering and Geomatics, Southwest Petroleum University, Chengdu, China

**Keywords:** Flexible rockfall barrier, Support rope, Tension force, Propagation distance, Attenuation coefficient, Natural hazards, Civil engineering, Mechanical engineering

## Abstract

This paper proposes a prediction method for the tension force of support ropes in flexible rockfall barriers. The method is based on two full-scale model tests with an impact energy of 3000 kJ, as well as 36 set numerical models featuring varying lengths and impact energies. From the results of full scale tests and numerical models, it is inferred that the tension force at the end of the support rope is significantly less than that at the point of impact, exhibiting an approximate Gaussian attenuation distribution with propagation distance. To account for the attenuation of tensile forces in support ropes, a tensile attenuation coefficient is defined. Through comparative analysis of data obtained from 36 models with varying impact energies and propagation distances, the average attenuation coefficient for the upper support rope is determined to be approximately 0.7, while the average coefficient for the lower support rope is around 0.8. Utilizing the least squares method, a prediction method for the tension force of support ropes in flexible rockfall barriers is established. This method takes into account both the propagation distance and impact energy, enabling accurate predictions of the tensile behavior of the ropes under different conditions. This prediction model provides valuable insights for engineers in the design and optimization of these flexible barriers for rockfall mitigation.

## Introduction

**Figure 1:**
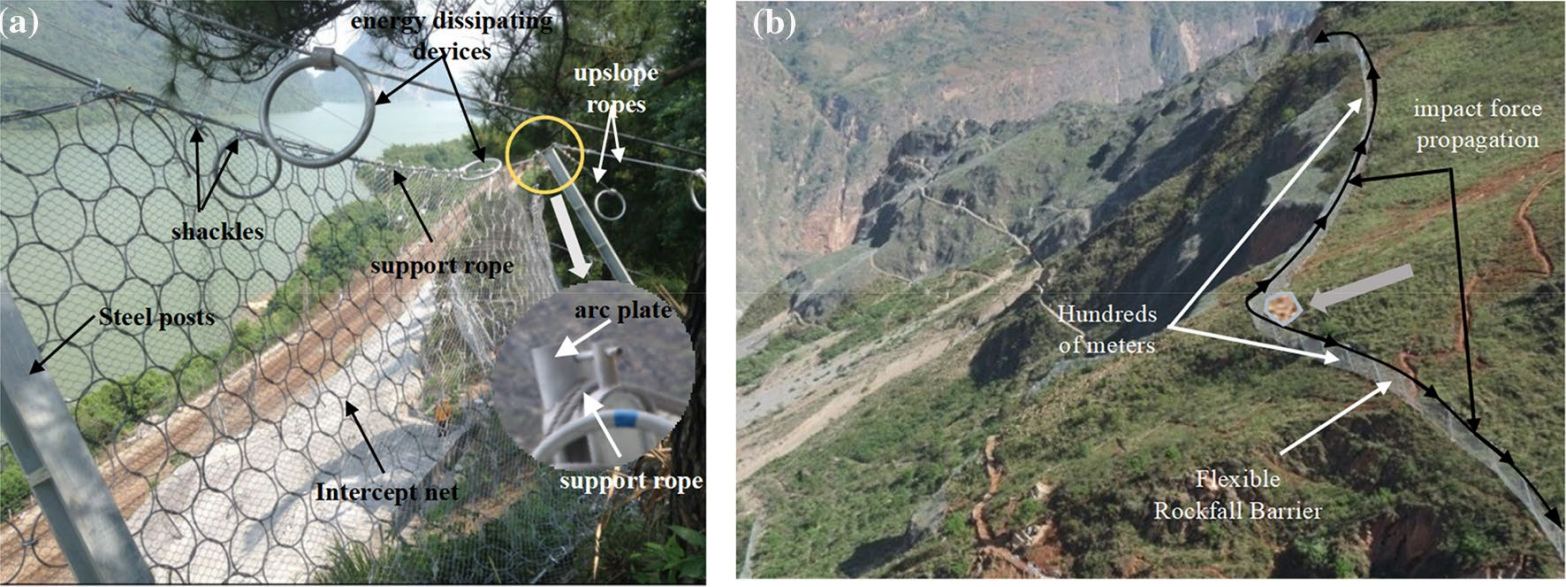
Component of flexible rockfall barrier.

Flexible rockfall barriers are one of the most common protection measures for falling rock disasters^1,2^, which primarily consist of interception structures(wire ring net), support structures(steel posts), and connection components(support ropes, upslope anchor ropes and energy dissipating devices)^3^. When a falling rock impacts the barrier, the wire ring net changes from a relaxed state to a tensile state, and the force is transferred to the support ropes. Once the force reaches a certain limit, the energy dissipating devices connected to the steel wire ropes start to deform. Through the large deformation of the wire ring net and the yield energy dissipation of energy dissipating devices, the energy of the falling rock is dissipated, successfully intercepting it^4^. In the effort of the wire ring net and the elongation of energy dissipating devices, the slippage of the support rope can reach several meters or even 10 m^5^. During the sliding process, the support ropes are mainly in contact with two components: one is the wire ring net , which is connected via shackles to create a slippery boundary, ensuring that the wire ring net can free slide on the support ropes, the other is the arc plate, which is set at the end of steel post to increase the bending radius, ensuring the support rope slide smoothly^6^ (Fig. 1a). Notably, strong nonlinear friction occurs at each contact place. Meanwhile, a flexible rockfall barrier typically consists of tens to hundreds of meters, contact effects are spread throughout the support rope, causing the tension of the support rope may attenuate with length (Fig. 1b).

**Figure 2:**
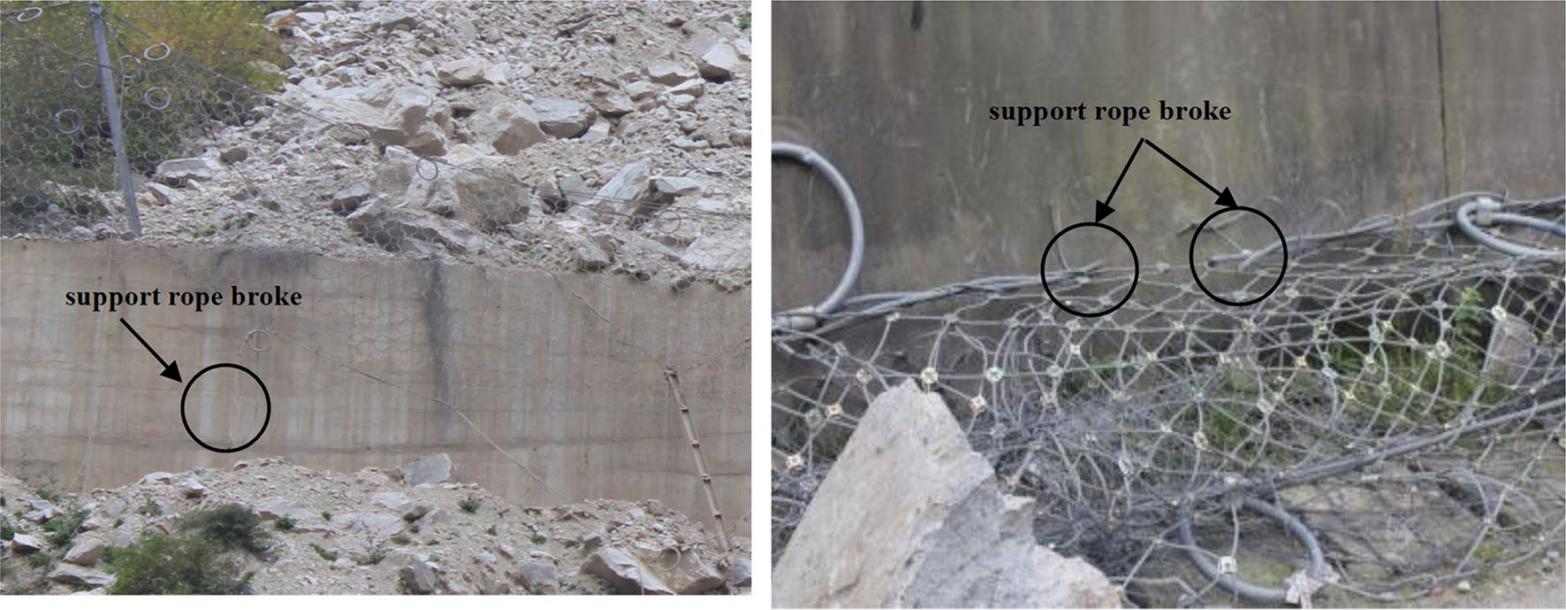
Broken support rope.

Usually, a large number of energy dissipating devices are installed on the support rope^7,8^. On the one hand, the energy dissipating devices provide deformation space for the support ropes, and on the other hand, their energy consumption accounts for more than half of the entire system^9^. Typically, they are positioned near the anchor points where near the end of support ropes^10^. Due to the attenuation characteristics of the support ropes, the tension transmitted to the end is far less than the tension force at the impact point, when the tension transmitted to the energy dissipating devices less than its starting force, which may not be activated. So, the impact energy cannot be dissipated, and the tension of the support rope increases quickly, in the end, causing the support rope to break and the entire system collapsed^11,12^ (Fig. 2).

To ensure that the flexible rockfall barrier can be used normally in practical applications, numerous scholars have conducted studies in view of the rockfall impact and the dynamic response of the system. For instance, optimized the system's structural layout to enhance the anti-impact ability of the barrier^13,14^. Studied the impact of rockfall at different incident angles, different places, different shapes and different friction coefficients on the dynamic response of the system^15–17^. Investigated the influence of impact energy and momentum change on the impact dynamic response of the system and proposed a highly nonlinear and dynamic numerical calculation method^18,19^.Some scholars examined the slip amount of the support rope under different rockfall impact scenarios. Some scholars have carried out full-scale impact tests of protective systems with different component specifications and different connection modes to reveal the basic mechanical behavior of protective structures^20–22^. Yu et al.^23^ built an overall model using discrete elements, and compared the slip and tension of the support rope using single impact and continuous full scale tests and numerical simulations. Dugelas et al.^24^ proposed a buffer mechanics model based on deflection control, for predicting the maximum deflection of the system. These studies propel the development of impact dynamics in flexible protective systems.

Nevertheless, research on the attenuation of cable forces of supporting ropes is insufficient, and the mechanical characteristics of supporting ropes of flexible rockfall barriers have still remained unknown. Previous studies have only constructed calibration tests to obtain the empirical values of safety factors of supporting ropes so that they cannot be extended to all engineering projects. Due to the limitation of measurement technology, in fact, the maximum cable force of the supporting rope has not been measured in tests up to now. Most existing research has only focused on the tension of local wire rope segments and the deformation of these rope segments, few studies have investigated the distribution and attenuation law of tension throughout the entire wire rope. Consequently, researching the tension force attenuation law of support ropes possesses practical significance for the development and application of flexible rockfall barriers.

## Analytical model for impact force transmission in support rope

**Figure 3:**
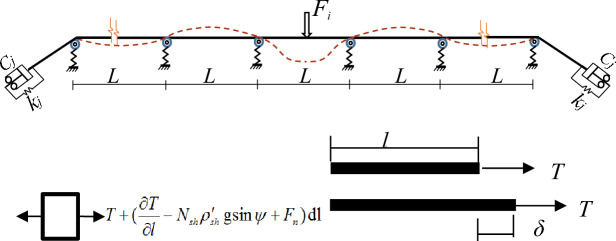
Structure and wire rope micro-element model.

A mechanical model that simplifies the support rope and its connecting structure has been established, where the impact force exerted by the wire ring net on the support rope is transformed into a pulling force on the energy dissipating device attached to it. By employing the Duhamel integral, the longitudinal motion equation of the infinitesimal element of the support rope has been derived, which is Eq. (1) and is illustrated in Fig. 3.

1 $$u(t) = \frac{1}{m\omega }\int\limits_{0}^{t} {F(t)e^{ - \mu \omega (t - \tau )} \sin \omega (t - \tau )d\tau }$$

To investigate the tension force waves in the support rope, the displacement *U* was taken as the unknown function and introduced the concept of longitudinal wave velocity *C*, as given by Eq. (2):

2 $$C = \left( {\frac{E}{{\rho_{sh} }}} \right)^{\frac{1}{2}}$$

By substituting Eq. (2) into Eq. (1) and performing the derivation, the governing equation for longitudinal wave propagation in the support rope is derived as Eq. (3):

3 $$\frac{{\partial^{2} u}}{{\partial^{2} t}} - C^{2} \frac{{\partial^{2} u}}{{\partial^{2} l}} - C^{2} \tau \frac{{\partial^{2} u}}{{\partial^{2} l\partial t}} = 0$$

where $\tau$ is the delay time. Due to the existence of viscous term, the harmonic solution is used to solve Eq. (3), resulting in the following expression: Eq. (4).:

4 $$\omega^{2} = C^{2} \mu^{2} + iC^{2} \tau \mu^{2} \omega$$

Letting $\mu = \alpha + i\beta$ in Eq. (4) , ignoring the unreasonable roots, then achieved:

5 $$\alpha^{2} { = }\frac{{\omega^{2} (\sqrt {1 + \omega^{2} \tau^{2} } { + }1)}}{{2C^{2} (1 + \omega^{2} \tau^{2} )}}$$

6 $$\beta^{2} { = }\frac{{\omega^{2} (\sqrt {1 + \omega^{2} \tau^{2} } - 1)}}{{2C^{2} (1 + \omega^{2} \tau^{2} )}}$$

Thus, the solution to Eq. (1) can be derived as Eq. (7):

7 $$U_{(t)} = Ae^{ - \beta } e^{i(\omega t - \mu l)}$$

According to^25,26^, considering that the wire rope tension is comprised of a static tension component and a dynamic tension effect, the functional relationship between the wire rope tension *T*, displacement *U*, and its derivatives is derived by combining Eq. (1) and Eq. (2):

8 $$T_{(x,t)} = N_{sh} \left[ {E + \eta \frac{\partial }{\partial t}} \right]\frac{{\partial u_{(x,t)} }}{\partial x}$$

where *η* is the viscous damping coefficient. Equation (7) and Eq. (8) indicate that the *β* term in the complex value *u* represents the exponential decay of the impact force wave with increasing propagation distance *L*. This decay behavior varies with frequency. For low-frequency waves, the $\beta$ is proportional to ${\omega }^{2}$. the tension wave exhibits a time lag due to the viscous damping term, resulting in attenuation of the tension in the wire rope subjected to shock wave transmission.

## Tension distribution in the support ropes

### Full-scale test

**Figure 4:**
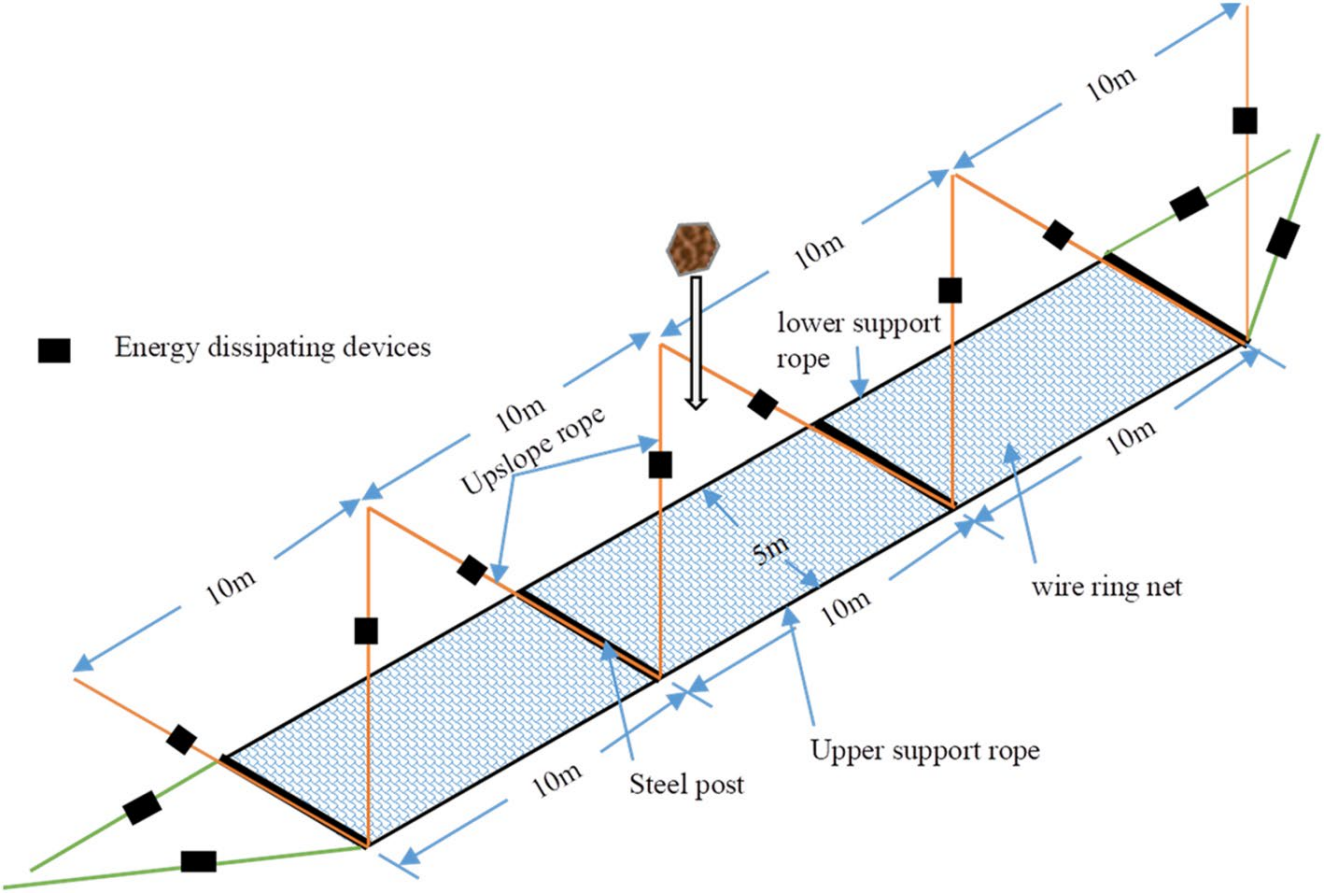
Test model and parameters.

The tests were carried out at the Southwest Jiaotong University Geological Disaster Test Site (Chengdu, Sichuan, China). The test model has three spans, each spanning is 10 m, and the wire ring net is in a horizontal state. A total of four steel posts, each with a spacing of 10 m and an elevation of 5 m, are set up to support the wire ring net. (Fig. 4). A gantry crane was used to lift the test block weighing 9400 kg to a height of 32 m from the wire ring net, with the impact position being the center of the mid-span, along with the impact energy of 3000 kJ. The specifications for each component of the test are listed in Table 1. Two full-scale models were constructed, with the only difference between Model 1 and Model 2 being the configuration of the energy dissipation devices.

**Table 1:** Specifications of the model components.

Component name	Model 1	Model 2
Wire Ring net	R16/3/300	R16/3/300
Upper/lower support rope	3Φ20	3Φ22
upslope anchor rope	2Φ22	2Φ22
Steel post	HW250 × 250 × 8 × 12	HW250 × 250 × 8 × 12
Energy dissipator on the support rope	Device1	Device2
Energy dissipator on the upslope anchor rope	Device1	Device3

### Numerical simulation

To accurately simulate the dynamic behavior of the flexible rockfall barrier, the commercial software LS-DYNA was adopted. In this section, the details of the numerical modeling technology of the main components and connections of the flexible rockfall barriers based on LS-DYNA are described.

#### Modeling of the components

**Figure 5:**
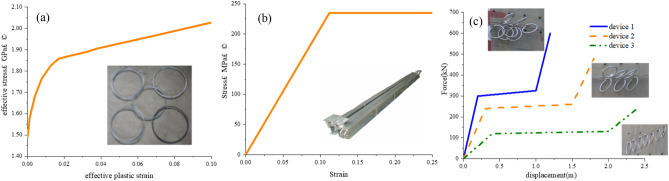
Constitutive model adopted in the numerical simulation.

Wire-ring net is modeled as an interconnected collection of rings, with each ring connected to four others using an automatic contact algorithm. The rings are constructed from super-strength steel wires with a diameter of 3 mm and a yield strength of 1.770 MPa. The beam element and a piecewise elastoplastic material model are used to describe the element's behavior. This behavior is defined by both the cross-sectional area and the uniaxial stress–strain relationship (Fig. 5a).

Steel posts play a crucial role in maintaining the overall integrity of the structure. As the posts were made of structural steel Q235, they could be accurately modeled using beam elements with an ideal elastic–plastic stress–strain curve, as shown in Fig. 5b. Therefore, the elastic modulus *E*s was 210 GPa, Poisson's ratio was 0.3, yield stress was 235 MPa, yield strain was 0.112%, and failure strain was 26%. In the numerical model, the boundary conditions of the post ends were assumed to be hinged support.

Steel wire ropes were modeled by beam elements with cable material type, so the tension-only characteristic was successfully modeled. The elastic modulus *E*s was 120 GPa, the Poisson’s ratio was 0.3, the yield stress was 1770 MPa, and the failure strain was 0.06.

Energy dissipating devices were modeled using nonlinear springs, and the force–displacement relationship was defined based on a group of tests. For Device 1, the starting force was 300 kN, with an energy consumption of 300 kJ. For Device 2, the starting force was 250 kN with an energy consumption of 375 kJ. For Device 3, the starting force was 120 kN with an energy consumption of 225 kJ (Fig. 5c).

#### Modeling of contact

The numerical model employed the automatic contact technique based on the symmetric penalty function method. An automatic beam-to-surface contact algorithm was used to simulate the contact behavior between the test block and the wire-ring net, with a dynamic friction coefficient of 0.2 specified between these elements. To reduce computational time, the block was positioned at the center of the middle functional module. The initial velocity of the block was defined based on the impact energy as 25m/s.

The support rope is connected to the wire-ring net by shackles, which were modeled as rigid elements. The guided-cable relationship was employed between the shackles and the support rope to allow for sliding during the impact process. Additionally, the support ropes can slide along the post saddle, which was modeled using sliding cable elements. A friction coefficient of 0.15 was set between the support rope and the post saddle.

### Impact process of the full-scale impact test and the numerical simulation

The results of the two experiments were significantly different. In the Model 1 test, the support rope broke, resulting in the test block falling. By contrast, in the Model 2 test, the system successfully intercepted the test block.

**Figure 6:**
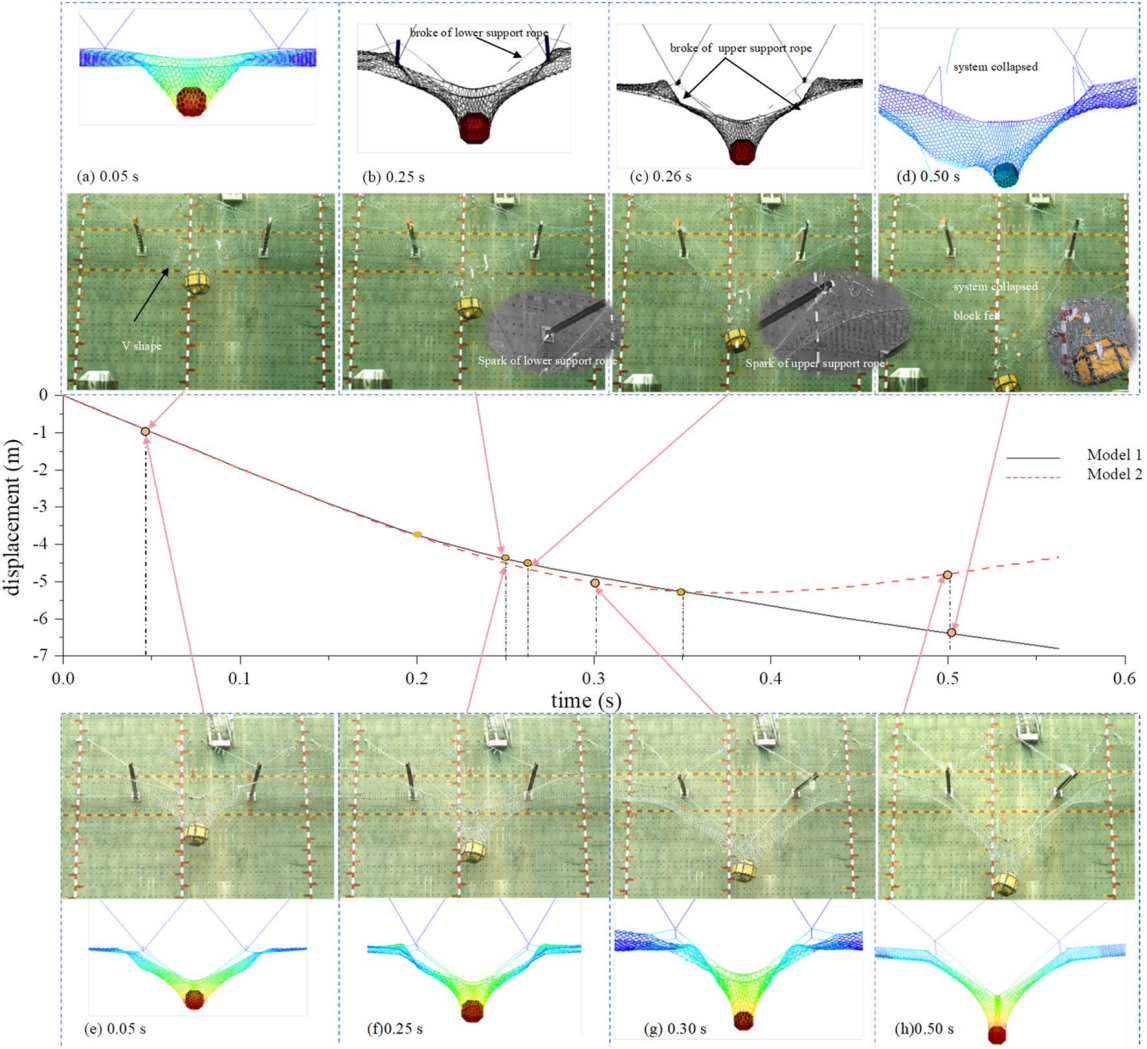
The impact process of Model 1 and Model 2.

During the first 0.25 s of the tests, the working state of the two models was basically the same. Upon impact with the test block, the wire-ring net initially underwent a V-shaped deformation, which was followed by a funnel-shaped deformation (Fig. 6a, e). At 0.25 s in Model 1 test, there were obvious sparks caused by friction at the contact point between the mid-span steel post and the steel wire rope. The lower support rope broke first (Fig. 6b), and the upper support rope broke 0.01 s later (Fig. 6c). In the Model 2 test, at 0.25 s, the support rope slid smoothly, and the energy dissipating devices on the support rope successfully initiated (Fig. 6f). Additionally, the energy dissipating devices on the upslope anchor rope effectively initiated, make sure the system cooperated and successfully intercept the test block (Fig. 6h).

After the test, it was found that in the Model 1 test, the steel post pin shaft of the central steel was seriously worn, and visible scratches were formed at the point where it contacted the support rope (Fig. 6d). The energy dissipating device on the upslope anchor rope was not activated, and the elongation length of the energy dissipating device on the support rope was minimal. However, in model 2, the energy dissipating devices on both the upslope anchor ropes and support ropes were fully elongated (Fig. 6g).

Further examination indicated that in Model 1, the funnel-shaped deformation of the ring net did not fully developed before the support rope fractured, and then the vertical force transferred by the horizontal support rope to the end of the impacted span's steel post was insufficient, leading to hard to start the energy dissipating devices of the upslope anchor rope. This blocked the net's impact deformation and made a significant tension increase in the support rope at the steel post. Due to the tension wave's transfer effect, there is a delay in the tension at the end of the wire rope, as a result, the energy dissipating devices at the end do not activate, and the support rope of the impacted span is fractured.

#### Tension of the wire rope

**Figure 7:**
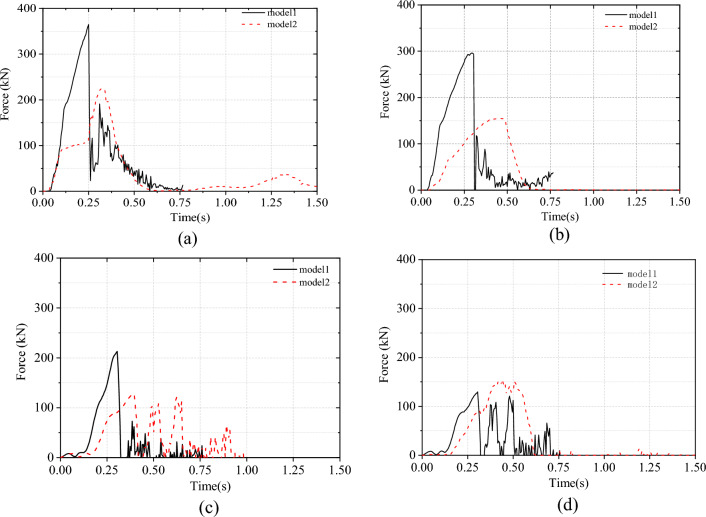
Comparison of the tensile force of steel wire ropes.

The tension of the wire rope at the end of Models 1 and 2 is individually measured and is presented in Fig. 7. It is evident that the tension of the support rope in Model 1 is higher than that in Model 2. At 0.25 s, the tension of the support rope in Model 1 increases sharply, with the lower support rope rapidly reaching a tension of 364 kN (Fig. 7a). Following the fracture of the lower support rope at the steel post saddle, there is a rapid decrease in steel wire rope tension. The breaking force of two steel wire ropes with a diameter of 22 is 504 kN^27^, indicating that the steel wire rope at the steel post saddle now has a tension greater than 504 kN. Subsequently, due to the broken of the lower support rope, all loads were transferred to the upper support rope, resulting in its immediate failure. The maximum tension supported by the upper support was 293 kN (Fig. 7b). In Model 2, both the upper and lower support ropes had significantly reduced peak tensions; specifically, the upper support rope had a peak tension of 152 kN and the lower support rope had a peak tension of 225 kN. In Model 1, there was a sharp increase in the tensile force of upslope anchor rope 3 due to an increase in support rope tension. However, in Model 2, this increase was less significant due to a coordinated change in the system. The results for upslope anchor rope 4 were similar in both models, with a force of only 150 kN (Fig. 7c, d).

#### Longitudinal distribution of the tension of support rope

**Figure 8:**
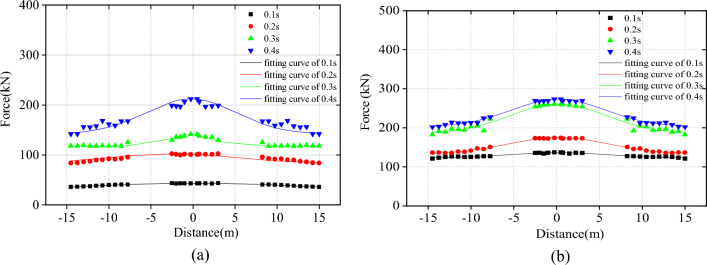
Longitudinal distribution of the tension force of support rope.

Based on Model 2, the impact point is taken as the coordinate origin to investigate the longitudinal distribution of the tension of the upper and lower support ropes at different moments (Fig. 8). As time progresses, the tension in the support rope gradually increases, peaking at 0.4 s. Notably, the tension in the lower support rope surpasses that of the upper support rope. The tension in the upper support rope experienced a significant increase between 0.2 s to 0.3 s, while the lower support rope's tension significantly increased between 0.3 s to 0.4 s. The tension distribution of the support rope seems to follow a Gaussian distribution, as shown in Eq. (9), which clearly forms three regions. The tension is significantly higher in the impact span than that in the side span. At 0.4 s, the tension at the end of the upper support rope amounts to 142.67 kN, which is 32.9% less than the tension at the impact point's 212.66 kN; and at the end of the lower support rope, the tension is 202.05 kN, experiencing a 26.2% reduction from the impact point's tension 273.76 kN.

9 $$F = y_{0} + A*\exp \left( { - \frac{{\left( {x - x_{c} } \right)^{2} }}{{2w^{2} }}} \right)$$

where *F* is the tension of the support rope, *x* is the longitudinal coordinate, *x*_*c*_ is the position coordinate of the symmetry axis,* y*_*0*_, and A and *w* are the different parameters corresponding to each moment, the parameter values are shown in Table 2.

**Table 2:** Parameters in the fitting formula corresponding to each moment.

	Upper support rope				Lower support rope			
	0.1 s	0.2 s	0.3 s	0.4 s	0.1 s	0.2 s	0.3 s	0.4 s
*y*_*0*_	11.75	65.73	118.90	141.72	123.23	132.81	185.29	202.46
*A*	31.58	35.83	31.40	62.60	13.01	41.96	75.81	70.45
*x*_*c*_	0.25	0.25	0.23	0.23	0.24	0.24	0.22	0.23
*w*	19.94	12.47	2.56	0.72	5.71	5.92	5.64	5.52

### Influence of Elastic modulus of support rope

Due to variations in the weaving techniques and compositions of support ropes, slight differences are exhibited in their elastic modulus. According to the literature^27^, the elastic modulus of steel wire rope is between 66 and 150 GPa.To investigate the influence of the elastic modulus of support rope in flexible rockfall barriers, take model 2 as the standard model, and the other five models only change the elastic modulus of the steel wire rope, the elastic modulus is taken as 60 GPa, 80 GPa, 100 GPa, 150 GPa and 180 GPa respectively, other structural parameters remain unchanged. The impact energy remained constant at 3000 kJ for all models.

**Figure 9:**
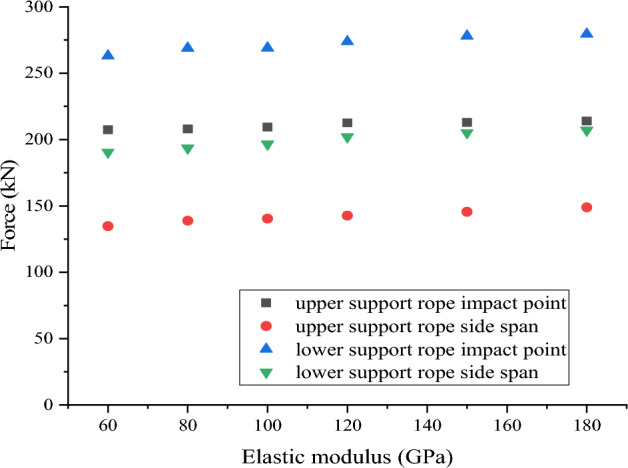
Influence of elastic modulus of support rope.

The calculation results as shown in Fig. 9 indicate that with the increase of elastic modulus, the tensile force of the support rope slightly increases. As the elastic modulus of the support rope increases from 60 GPa to 180 GPa, the tensile force at the impact point of the upper support rope increases by only 3.2%, while the tensile force at the impact point of the lower support rope increases by 5.7%, the tensile force at the side span of the support rope increases by about 8.0%. It is worth noting that in each case, the ratio of the support rope tension at the end to the wire rope tension at the impact point is basically consistent, with the upper support rope ratio ranging from 0.66 to 0.68 and the lower support rope ratio ranging from 0.72 to 0.74. Therefore, the change in elastic modulus has a relatively little influence to the tension attenuation characteristics .

## Influence of propagation distance

To investigate the influence of propagation distance on multi-span flexible rockfall barriers, six models with different numbers of spans (3, 4, 5, 6, 7, and 9) were established. The impact energy remained constant at 3000 kJ for all models. In each model, the span length was fixed at 10 m, the protective height was 5 m, and the energy dissipating devices were installed at a distance of 7.5 m from the side post. The impact position was at the center of the axis of symmetry for odd-numbered span structures, while for even-numbered span structures, it was at the center of the adjacent spans to the left of the axis of symmetry. The propagation distance was defined as the distance from the impact position to the end point of the wire rope. For even-numbered span models, the propagation distances on both sides were different; therefore, the average distance on both sides was taken. The propagation distances were 22.5 m, 27.5 m, 32.5 m, 37.5 m, 42.5 m, and 52.5 m.

### Impact deformation

**Figure 10:**
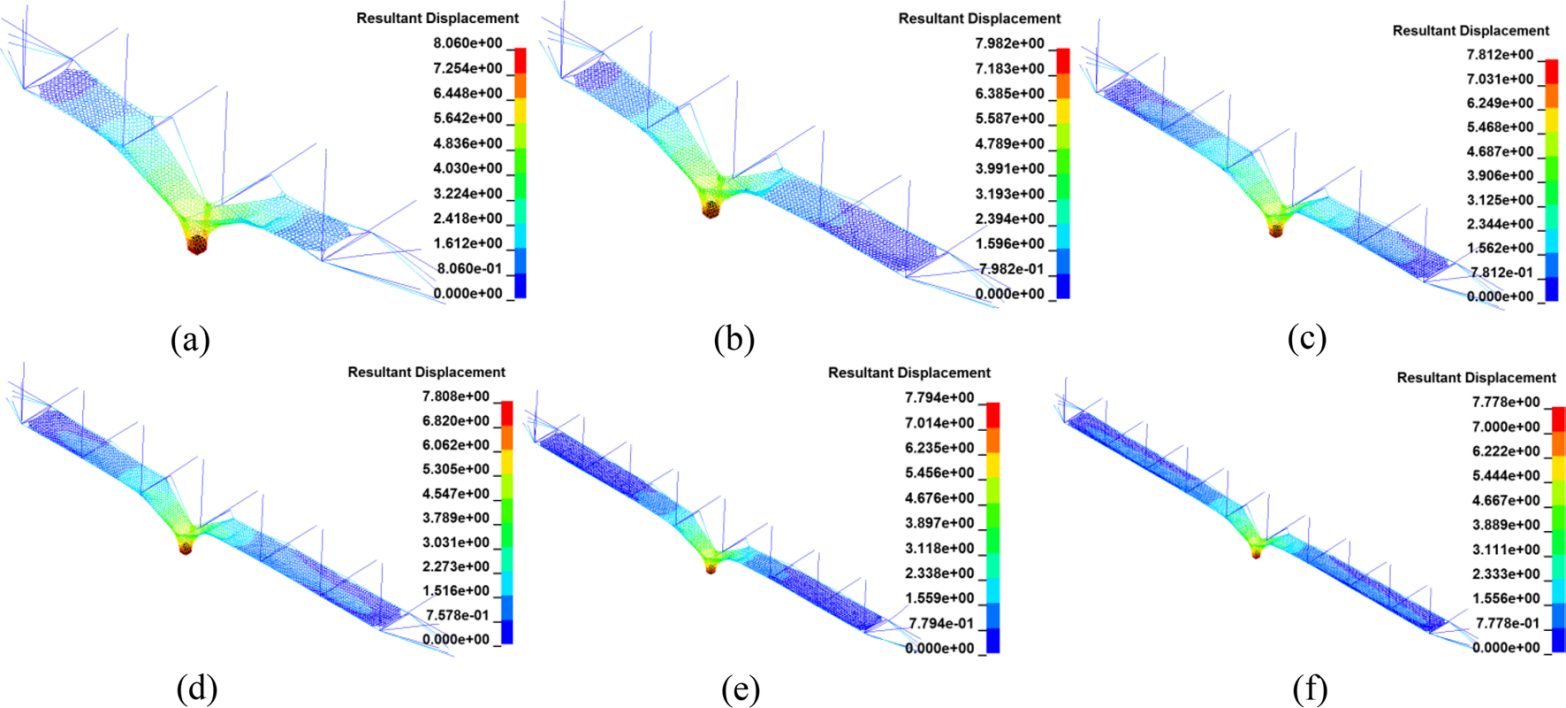
Displacement of the multi-span models.

After impact, each structure effectively intercepted the test block, and the impact deformations are shown in Fig. 10. As the number of spans increased, the displacement of the rockfall decreased slightly, from 8.06 m for the model with three spans to 7.78 m for the model with nine spans, which is a decrease of about only 3%. Therefore, the effect of propagation distance on impact deformation can be ignored.

### Tension attenuation

According to the analysis in Section "Tension distribution in the support ropes", the tension is significantly lower at the end of the wire rope compared to the impact point of the tension. To further investigate this phenomenon, the tension attenuation coefficient was defined as the ratio of the support rope tension at the end to the wire rope tension at the impact point, as shown in Eq. (12).

12 $$\beta = \frac{{F_{end} }}{{F_{imp} }}$$

where ${F}_{imp}$ is the tension force at the impact point of the support rope and ${F}_{end}$ is the tension force at the end of the support rope.

The tension distribution and attenuation coefficient of the models with different span numbers are presented in Table 3. As the propagation distance increases, the tension at the impact point and the end of the support rope decreases correspondingly. Notably, the tension at the impact point of the upper support rope decreases most obviously, from 212 kN to 172 kN, representing a decrease of 18%. The tension in the other positions decreases by approximately 12%. At the same time, there is no obvious difference in the tension attenuation coefficient of the upper support rope, which is approximately 0.67. The tension attenuation coefficient of the lower support rope increases from 0.738 to 0.774. Although the tension at the impact point and the end of the lower support rope decreases, the tension attenuation rate is evidently greater at the impact point of the lower support rope than that at the end of the rope, leading to a slight increase in the tension attenuation coefficient of the lower support rope.

**Table 3:** Tension force and tension attenuation coefficient in each case.

	Propagation distance /m	Upper support rope			Lower support rope		
		*F*_imp_/kN	*F*_end_/kN	$\beta$	*F*_imp_ /kN	*F*_end_ /kN	$\beta$
Case 1	22.50	212.66	142.67	0.671	273.756	202.048	0.738
Case 2	27.50	192.19	129.19	0.672	259.679	199.36	0.768
Case 3	32.50	188.58	126.06	0.668	255.304	196.966	0.771
Case 4	37.50	176.01	124.31	0.706	250.145	196.154	0.784
Case 5	42.50	174.05	123.08	0.707	245.483	191.709	0.781
Case 6	52.50	172.67	120.27	0.697	234.22	181.384	0.774

## Law of tension attenuation of the support rope

In actual engineering applications, the protective energy of a flexible rockfall barrier can vary. To investigate the effects of different energy levels on the tension attenuation characteristics of the support rope, numerical analysis models were established with protection energy levels of 1000kJ, 2000kJ, 3000kJ, and 5000kJ. The system configuration for each energy level is provided in Table 4. For each protective energy level, six groups of different propagation distances were investigated, and the impact positions were consistent with the models of each span as previously mentioned.

**Table 4:** System configuration for models with different protection energy level.

	Wire ring Net	Upper/Lower support rope	Upslope anchor rope	Steel post	Energy dissipating devices on the support rope	Energy dissipating devices on the upslope anchor rope
1000kJ	R12/3/300	2Φ20	1Φ18	HW200 × 200x8 × 12	Device 3	Device 3
2000 kJ	R12/3/300	3Φ20	1Φ22	HW250 × 250x8 × 12	Device 3	Device 3
3000kJ	R16/3/300	5Φ22	2Φ22	HW300 × 300x10 × 15	Device 2	Device 3
5000kJ	R19/3/300	8Φ22	4Φ22	HW300 × 300x10 × 15	Device 1	Device 2

### Tension distribution of Support rope

**Figure 11:**
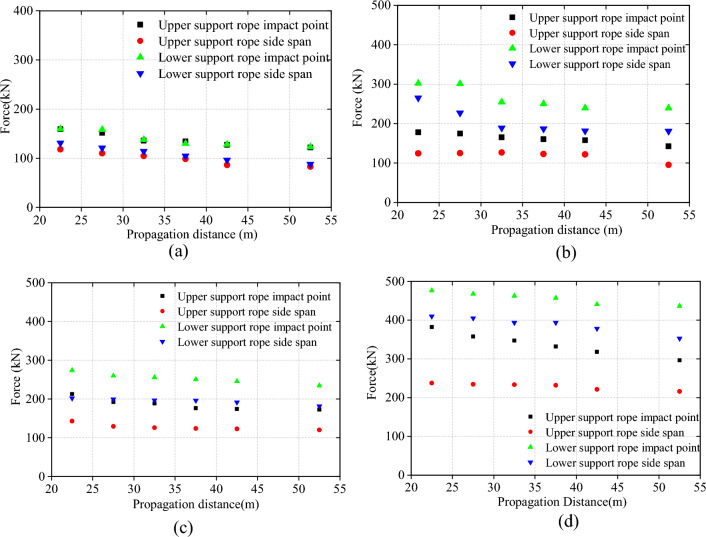
Tension force distribution of the support rope.

The tension force of the upper and lower support ropes at the impact points and end points of each model were extracted and plotted in Fig. 11. The figure clearly shows that in the same model, the tension force at the impact point of the support rope is higher than that at the end, indicating a clear trend of tension attenuation along the rope.

Additionally, as the impact energy increases, the difference between the tension force at the end and the tension force at the impact point also increases. For example, in the 1000 kJ model, the tension force at the impact point is basically the same as that at the end. However, in the 5000 kJ model, the tension force at the impact point of the lower support rope is about twice the end force value.

### Tensile attenuation coefficient of the support rope

**Figure 12:**
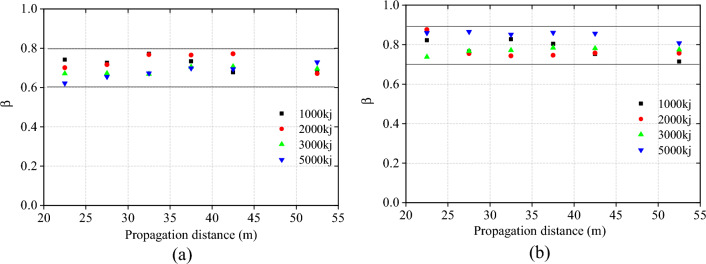
Tension attenuation coefficient of each model.

The tension attenuation coefficient *β* of each model was computed using Eq. (7) and plotted in Fig. 12. The figure clearly shows that the tension attenuation coefficient *β* for the upper support rope is concentrated between 0.6 and 0.8, while the tension attenuation coefficient *β* for the lower support rope is concentrated between 0.7 and 0.9. These findings suggest that there is a consistent trend in the tension attenuation coefficient *β* for both the upper and lower support ropes. The average value of the tension attenuation coefficient *β* for the upper support rope is approximately 0.7, while the average value for the lower support rope is approximately 0.8.

### Tension prediction

Based on the 36 models with different impact energies and different propagation distances in Section "Tension distribution in the support ropes" and Section "Influence of propagation distance", the tensions of the support ropes at the impact point are extracted respectively. Utilizing the least squares method, a mathematical model is fitted to describe the tensile force value at the impact point of both the upper and lower support ropes $T_{imp - \max }$. This model considers the impact energy ($E_{k}$) and the propagation distance of the system (*L*) as parameters. ( Eq. 13)

13 $$T_{imp - \max } = \alpha_{1} L^{{\alpha_{2} }}$$

For the upper support rope:

14 $$\alpha_{{{1 - }up}} = {18}{\text{.85}} \times \left( {\frac{{E_{k} }}{1000}} \right)^{2} - {34}{\text{.52}} \times \left( {\frac{{E_{k} }}{1000}} \right) + 4{36}{\text{.02}}$$

15 $$\alpha_{{{2 - }up}} = - {0}{\text{.004}} \times \left( {\frac{{E_{k} }}{1000}} \right)^{2} + {0}{\text{.07}} \times \left( {\frac{{E_{k} }}{1000}} \right) - {0}{\text{.39}}$$

For the lower support rope:

16 $$\alpha_{{{1 - }low}} = {15}{\text{.89}} \times \left( {\frac{{E_{k} }}{1000}} \right)^{2} - {18}{\text{.96}} \times \left( {\frac{{E_{k} }}{1000}} \right) + {519}{\text{.25}}$$

17 $$\alpha_{{{2 - }low}} = - {0}{\text{.004}} \times \left( {\frac{{E_{k} }}{1000}} \right)^{2} + {0}{\text{.07}} \times \left( {\frac{{E_{k} }}{1000}} \right) - {0}{\text{.42}}$$

where $E_{k}$ is the impact energy, *L* is the propagation distance, and $\alpha_{1}$、$\alpha_{2}$ are the correlation coefficients.

### Cross-validation

To cross-verify the correctness of the formula, the test described in reference^13^ is selected for verification. The test model had three spans, the propagation distance was 22.5 m, and the impact energy was 5246 kJ. In the test, the measured tension forces of the upper support rope and the lower support rope were 390 kN and 450 kN, respectively. Combined with Eq. (13) to Eq. (17), the simulated tension forces of the upper support rope and the lower support rope were 359 kN and 413 kN, respectively. The prediction results are slightly less than the experiment results, with a maximum error of 8.2%, as shown in Table 5. It indicates that the prediction method has sufficient accuracy.

**Table 5:** Comparison of the prediction and experiment results.

	Literature^13^ (kN)	Prediction method (kN)	Error (%)
Upper support rope	390	359	7.9
Lower support rope	450	413	8.2

## Engineering application

**Figure 13:**
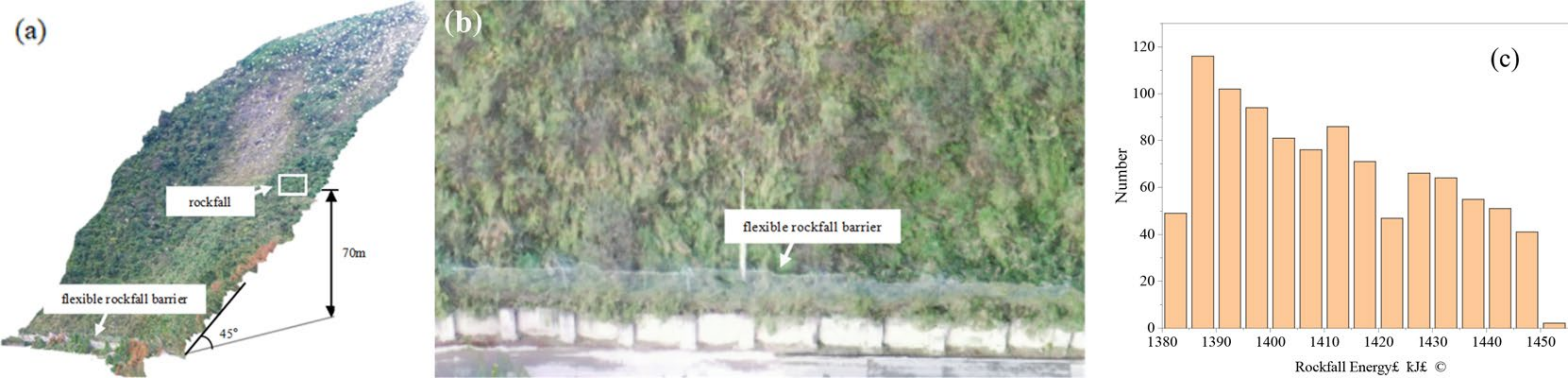
Locations of the rockfall sources and the flexible barrier.

A flexible rockfall barrier is proposed to be erected along a mountainous railway. After three-dimensional (3D) scanning of the mountain and a simulation of rockfall track in the early stage (Fig. 13a), the total length of the structure to be erected is 70 m, with each span of 10 m (Fig. 13b). Based on Rocfall software, using 1000 drop simulations, to calculate the energy of the impact of falling rocks on the flexible rockfall barrier (Fig. 13c), the energy range of falling rocks is 1380 kJ–1450 kJ, so the Service Energy Level (SEL) is 1500 kJ, according to EAD 340,059-00-0106 ^28^, the Maximum Energy Level (MEL) is 4500 kJ.

**Figure 14:**
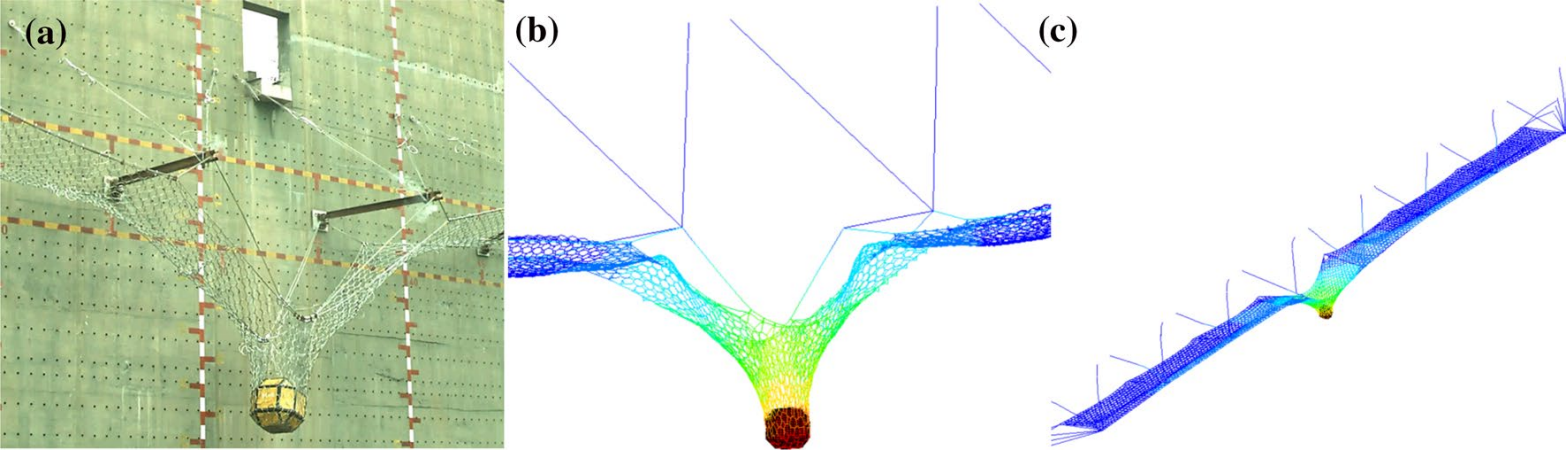
Test and numerical verification.

**Figure 15:**
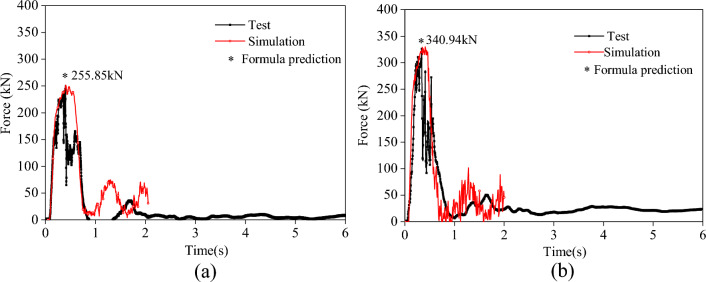
Comparison of support rope tension.

The specifications of each component in the system are consistent with the system configuration with a protection level of 5000 kJ mentioned in 5.2. To check the system configuration, full-scale verification tests were conducted. Due to site restrictions, the verification model only had three spans, and the verification model was completely consistent with other configuration parameters of the actual structure (Fig. 14a). Simultaneously established a numerical model consistent with the experimental model parameters (Fig. 14b) . At the same time, combined with Eq. (13) to Eq. (17), the tension of the support rope of the test verification model was estimated and compared with the results of the test and numerical simulation. As shown in Fig. 15 and Table 6, the prediction formula is in good agreement with the results of the numerical simulation and test. The prediction results are slightly larger than those of experimental and numerical simulation, with an error of 8%. The difference between the peak value of the internal force of the support rope obtained from the test and numerical analysis is small, but the internal force of the steel rope collected in the test fluctuates to a certain extent, while the time history of the internal force of the steel rope in the numerical analysis is relatively smooth. The reason is that the performance of the energy dissipating devices in practical applications is fluctuating, while the energy dissipating devices in the numerical analysis are an ideal working curve.

**Table 6:** Tension forces of support ropes in the 3-span model.

	Test (kN)	Simulation (kN)	Formula (kN)	Error (%)
End point of upper support rope	249.92	249.61	255.85	2.37
End point of lower support rope	325.08	326.01	340.94	4.88

Furthermore, a 7-span numerical model of the actual structure was established (Fig. 14c). Simultaneously using formulas to calculate the tension forces at the impact points and the end points of the support rope based on the actual structure. The formula calculation results also maintain good consistency with the numerical analysis results, with an error of 7% (Table. 7).

**Table 7:** Tension forces of support ropes in the 7-span model.

	Simulation(kN)	Formula(kN)	Error (%)
Impact point of the upper support rope	348.11	365.49	4.99
Impact point of the lower support rope	407.92	426.17	4.47
End point of the upper support rope	275.38	255.84	7.10
End point of the lower support rope	320.26	340.94	6.46

Based on the formula calculation results, design suggestions are given as follows, the maximum tension at the impact point of the supporting rope is 426.17 kN. Considering the dynamic amplification factor of 1.5 times, it is suggested that the breaking force of the steel wire rope should be at least greater than 426.17*1.5 = 639.22 kN. The minimum tension at the end of the support rope decreases to 250.84 kN. Considering the impulse effect of the starting force, it is suggested that the starting force of the support rope connection energy dissipating devices is less than 255.84*0.8 = 204.67kN. This provides a practical approach for the selection or design of flexible rockfall barriers.

## Conclusion

Based on full-scale tests and numerical simulation, this paper presents a prediction method for the tensile force of support ropes in a flexible rockfall barrier. the following conclusions can be drawn:

1. The tension force in the support rope exhibits notable disparities between the impact span and the other side spans. The tension peaks in the impact area and gradually decreases on both sides. When the impact location in the three-span model is the central point, the internal force distribution of the support rope seems to follow a Gaussian distribution.
2. When the impact energy remains constant but the propagation distance varies, the maximum displacement of the system remains relatively unchanged as the propagation distance increases. However, the tension attenuation is evident, with the tension at the end of the upper support line decreasing the most significantly, by 18%. At other positions, the tension reduction is approximately 12%. The minimum attenuation coefficient reaches 0.67. However, the elastic modulus of support ropes have little influence to the tension attenuation characteristics.
3. For structures with different impact energies and different propagation distances, the tensile force at the impact point of the support rope decreases in a power function as the propagation distance increases, and they linearly increase as the impact energy increases. Overall, the statistics reveal that the average attenuation coefficient of the upper support rope is 0.7, while the average value of the attenuation coefficient of the lower support rope is 0.8.
4. The prediction equations of the tension force in the support rope are derived through curve fitting. These formulas can be employed to estimate the tensile force in a flexible rockfall barrier and provide a basis for structural design.

It should be clarified that the findings from this study are based on specific types of flexible rockfall barriers. Further studies on additional factors that could influence the tension force of the barrier are recommended. For example, the impact angle of rockfall, multiple rockfall impacts and rock shape may be taken into account.

## Data Availability

The datasets used or analysed during the current study are available from the corresponding author on reasonable request.
